# A Robust [^18^F]-PSMA-1007 Radiomics Ensemble Model for Prostate Cancer Risk Stratification

**DOI:** 10.1007/s10278-024-01281-w

**Published:** 2024-09-30

**Authors:** Giovanni Pasini, Alessandro Stefano, Cristina Mantarro, Selene Richiusa, Albert Comelli, Giorgio Ivan Russo, Maria Gabriella Sabini, Sebastiano Cosentino, Massimo Ippolito, Giorgio Russo

**Affiliations:** 1 Institute of Bioimaging and Complex Biological Systems - National Research Council (IBSBC - CNR), Contrada, Pietrapollastra-Pisciotto, 90015 Cefalù, Italy; 2 Department of Mechanical and Aerospace Engineering, Sapienza University of Rome, Eudossiana 18, 00184 Rome, Italy; 3 National Laboratory of South, National Institute for Nuclear Physics (LNS-INFN), 95125 Catania, Italy; 4 Nuclear Medicine Department, Cannizzaro Hospital, 95125 Catania, Italy; 5 Ri.MED Foundation, Via Bandiera 11, 90133 Palermo, Italy; 6 Department of Surgery, Urology Section, University of Catania, 95125 Catania, Italy; 7 Medical Physics Unit, Cannizzaro Hospital, 95125 Catania, Italy

**Keywords:** Radiomics, [^18^F]-PSMA-1007 PET, Prostate cancer, Machine learning

## Abstract

The aim of this study is to investigate the role of [^18^F]-PSMA-1007 PET in differentiating high- and low-risk prostate cancer (PCa) through a robust radiomics ensemble model. This retrospective study included 143 PCa patients who underwent [^18^F]-PSMA-1007 PET/CT imaging. PCa areas were manually contoured on PET images and 1781 image biomarker standardization initiative (IBSI)-compliant radiomics features were extracted. A 30 times iterated preliminary analysis pipeline, comprising of the least absolute shrinkage and selection operator (LASSO) for feature selection and fivefold cross-validation for model optimization, was adopted to identify the most robust features to dataset variations, select candidate models for ensemble modelling, and optimize hyperparameters. Thirteen subsets of selected features, 11 generated from the preliminary analysis plus two additional subsets, the first based on the combination of robust and fine-tuning features, and the second only on fine-tuning features were used to train the model ensemble. Accuracy, area under curve (AUC), sensitivity, specificity, precision, and *f*-score values were calculated to provide models’ performance. Friedman test, followed by post hoc tests corrected with Dunn-Sidak correction for multiple comparisons, was used to verify if statistically significant differences were found in the different ensemble models over the 30 iterations. The model ensemble trained with the combination of robust and fine-tuning features obtained the highest average accuracy (79.52%), AUC (85.75%), specificity (84.29%), precision (82.85%), and *f*-score (78.26%). Statistically significant differences (*p* < 0.05) were found for some performance metrics. These findings support the role of [^18^F]-PSMA-1007 PET radiomics in improving risk stratification for PCa, by reducing dependence on biopsies.

**Supplementary Information:**

The online version contains supplementary material available at 10.1007/s10278-024-01281-w.

## Introduction

According to the World Health Organization (WHO), prostate cancer (PCa) is the second most frequent type of cancer in men, accounting for approximately 1.5 million diagnosed cases worldwide, and the third leading cause of cancer deaths in Europe [1]. During prostate screening, elevated prostate-specific antigen (PSA) levels indicate increased risk of PCa. However, PSA is organ-specific rather than tumor-specific, and the specificity of PSA in representing disease severity remains controversial. PSA is also abnormally elevated in many benign prostate diseases, leading to high missed diagnosis and misdiagnosis rates [2]. So, using PSA level as the sole discriminant for PCa risk stratification is not specific, resulting in many unnecessary prostate biopsies. Prostate biopsy is associated to the risk of hematuria, infection, pain, inflammation, and sepsis; its indication should be carefully evaluated [3]. In the case of transperineal or transrectal ultrasound–guided (TRUS) biopsies, the presence of PCa is confirmed through risk stratification based on the Gleason scoring (GS): a primary Gleason grade is assigned to the most prevalent histological pattern observed, while a secondary grade is assigned to the next-most frequent pattern, based on the microscopic structure and appearance of the cells. Consequently, GS relies on portions of prostate sampled through biopsy, neither characterizing its whole volume, nor cancer heterogeneity, decreasing the reliability of the final score.

Therefore, imaging modalities, such as whole-body magnetic resonance imaging (MRI), prostate-specific membrane antigen (PSMA), positron emission tomography/computed tomography (PET/CT), and PSMA–PET/MRI, were introduced for better cancer localization and staging, refining the discrimination between intermediate-risk and high-risk PCa [4–7]. PSMA is a transmembrane glycoprotein that is upregulated in PCa and its expression correlates with the degree of malignancy [8]. An accurate PCa staging helps to improve the correct treatment plan, reducing the risk of overtreatment. Furthermore, radiomics-based models trained with features derived from MRI, PET/MRI [9, 10], and PET [11–17] images showed their ability for the non-invasive characterization of PCa, specifically for the risk stratification prediction, primary PCa detection and biochemical recurrence prediction, potentially resulting in a tool for reducing overdiagnosis and overtreatment incidence. PSMA-PET showed excellent sensitivity and specificity for recurrent PCa and promising results in bone metastasis detection, especially when the [^68^ Ga]Ga radionuclide was used [18, 19]. However, its limitations, such as the short lifespan, suboptimal energies, and challenging production, motivated the consideration of [^18^F]-labelled analogues, and the [^18^F]-PSMA-1007 was individuated as the best radiotracer to overcome these issues [20]. Like other forms of medical imaging, reporting of PSMA-PET is prone to variability between readers and so there is significant interest in integrating artificial intelligence (AI) into medicine [21]**.**

In the context of prostate cancer imaging, most studies have traditionally relied on MRI rather than PET imaging, and radiomics applications involving different radiopharmaceuticals have been relatively limited. Specifically, the potential of [^18^F]-PSMA-1007 in radiomics has been underexplored, with far fewer studies utilizing this radiopharmaceutical compared to [^68^ Ga]Ga-PSMA-11 [22, 23]. In addition, many studies fail to provide comprehensive details on feature extraction parameters and model hyperparameters, which are essential for reproducibility. The omission of these critical elements impairs the ability to replicate findings and apply them in clinical settings [24]. In fact, despite the valuable insights radiomics offers into tumour biology and treatment response, the successful integration of PET radiomics into clinical practice is contingent upon overcoming specific challenges and limitations. The review [25] underscores the necessity of addressing these issues, particularly the need for standardized pre- and post-processing methodologies. Without these, the radiomics risks limited clinical applicability. To address these challenges, we propose a robust and reproducible radiomics pipeline tailored to PET imaging with [^18^F]-PSMA-1007. This pipeline ensures consistent feature extraction, enhances model reliability, and promotes the clinical translatability of radiomics analyses, ultimately enabling a more effective application of PET-based radiomics in oncology. Briefly, our approach aims to provide more reliable and clinically meaningful results in this way: (i) building a robust ensemble model based on radiomics, with the potential to improve discrimination between high- and low-risk patients; (ii) providing a comprehensive analysis of the stability of radiomics features; and (iii) identifying the most predictive subset of features and optimize model hyperparameter settings.

Leveraging an iterated initial analysis pipeline consisting of repeated training-test splitting and *k*-fold cross-validation, we managed to detect the most robust features, the pool of model hyperparameters and the best candidate models to build the ensemble model based on average performance. Then, from the initial pool of hyperparameters, we individuated their best combination and refined the subset of selected features maximizing the performance of the model. This study provides a robust and comprehensive radiomics method for developing machine learning ensemble models.

## Materials and Methods

### Patients

Patients who underwent [^18^F]-PSMA-1007 PET/CT imaging for PCa staging between October 2021 and April 2023 at the Cannizzaro Hospital of Catania (Italy) were recruited. Inclusion criteria were as follows: (i) confirmation of PCa by needle biopsy of the prostate, (ii) availability of PCa grade; and (iii) positive PET scans. The exclusion criteria were as follows: (1) patients referred for treatment prior to study enrolment, including surgery, chemotherapy, radiation therapy, endocrine therapy, or any other intervention; and (2) patients with a previous history of other cancers.

**Fig. 1:**
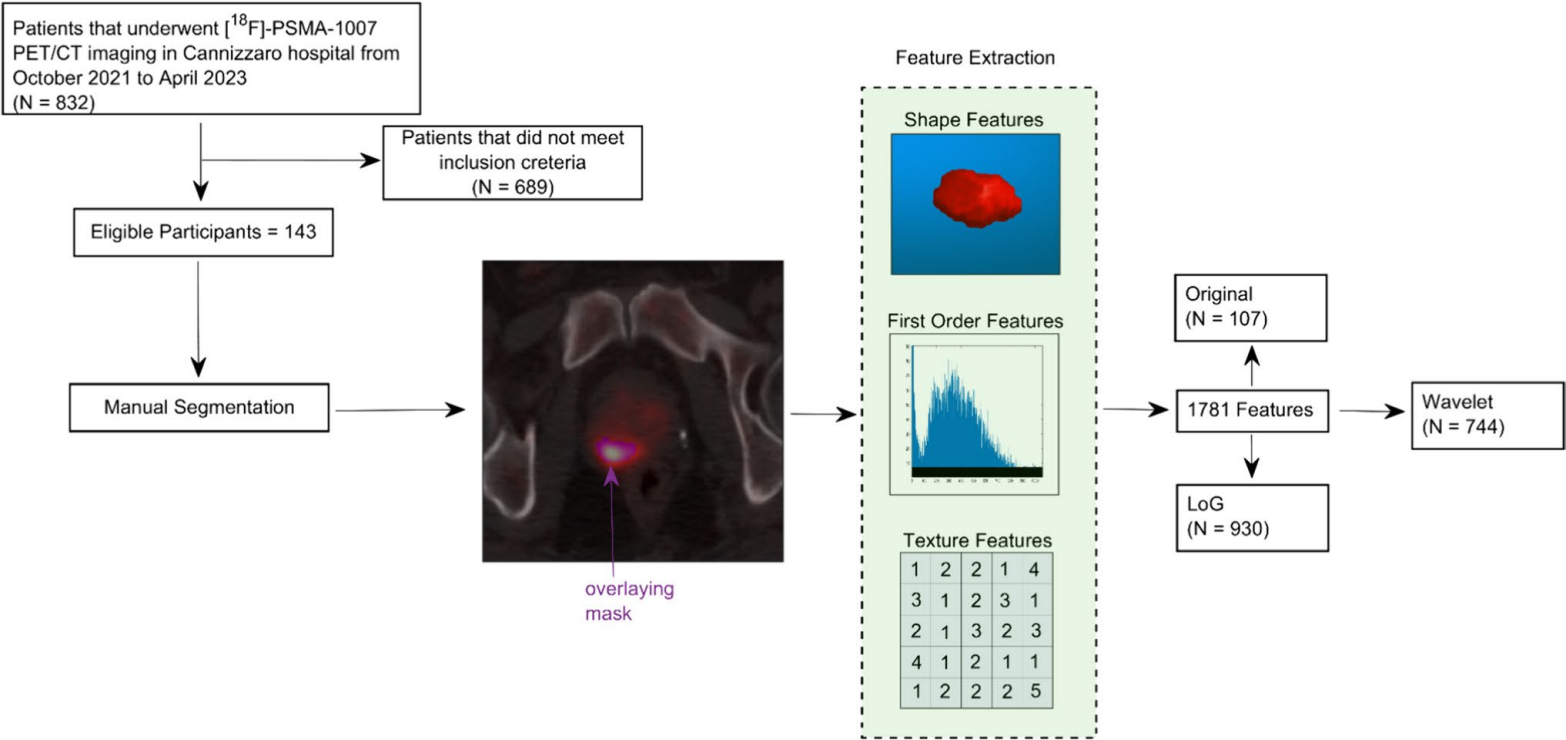
Flowchart showing the patients’ selection process and the radiomics workflow applied to the 143 PCa patients meeting the inclusion criteria. A purple mask overlays the PCa in PET/CT image. A total of 1781 radiomics features were extracted for each lesion

Among the PCa patients screened, 143 met the criteria and were used for analysis. See Fig. 1 for a flowchart showing the patients’ selection process.

Post-surgical grades were used to stratify the cohort into low-grade and high-grade PCa, as reported in the “Gleason grade analysis” section.

This retrospective study was conducted in accordance with the Declaration of Helsinki and was approved by the local ethics committee (REDIRECT Study, n.101/2022). Informed consent was waived due to the retrospective nature of the study.

### [^18^F]-PSMA-1007 PET/CT Imaging

Discovery 690FX&MOT (General Electric Milwaukee, WI, USA) and Biograph Horizon 4R (Siemens Knoxville, TN, USA) PET/CT scanners were used for image acquisition of 68 and 75 PCa patients, respectively.

For the first scanner, 16 row helical CT scan was used with the following conditions: tube voltage (140 kVp), tube current (800 mAmax). PET acquisition time (90 s), beds per scan (7–8). PET matrix (256 × 256), and CT matrix (512 × 512). PET voxel size (2.73 × 2.73 × 3.27 mm^3^), and CT voxel size (1.37 × 1.37 × 3.75 mm^3^).

For the second scanner, 16 row helical CT scan was used with the following conditions: tube voltage (130 kVp) and tube current (345 mAmax); PET acquisition time (90 s) and beds per scan (6–7); PET matrix (512 × 512) and CT matrix (512 × 512); the PET voxel size (1.45 × 1.45 × 3 mm^3^) and the CT voxel size (0.98 × 0.98 × 3 mm^3^).

PET scans were acquired according to the normal clinical practice, following the joint EANM and SNMMI procedure guideline for PCa imaging [26]. Specifically, examinations started approximately 120 min after the injection of approximately 4 MBq per kilogram body weight. PET image interpretation was conducted by two nuclear medicine physicians. The presence of intraprostatic focal and intense increased uptake of [^18^F]-PSMA-1007, with or without visible alterations on co-registered CT images, was considered positive for malignancy.

### Gleason Grade Analysis

The GS was obtained from the histological reports of the biopsies to which the patients were subjected, coming from the Pathological Anatomy Department. Since, prostate cancer aggressiveness is quantified using GS that is the sum of the grades (ranging from 1 to 5) assigned to the two primary areas of cancerous tissue taken by biopsy [27], such as follows:

$$\text{GS}={\text{grade}}_{\text{Area}1}+{\text{grade}}_{\text{Area}2}$$

where ${\text{grade}}_{\text{Area}1}$ is the grade assigned to the first area and the predominant grade, while ${\text{grade}}_{\text{Area}2}$ is the grade assigned to the second area, and given that:

- GS ≤ 6: Indicative of low-aggressive prostate tumours with low metastatic potential
- GS = 7: Indicative of tumours of intermediate aggressiveness
- GS between 8 and 10: high-aggressive tumours with high metastatic potential

Patients with GS 3 + 3 or 3 + 4 were assigned to the low–risk group, while patients with GS 4 + 3, 4 + 4, 4 + 5, or 5 + 5 were assigned to the high–risk group.

### Image Segmentation and Feature Extraction

Segmentation was performed by an experienced nuclear medicine physicist (C.M author), which manually segmented prostate cancer areas slice-by-slice. In cases of uncertainty, author M.I. (head of the NM department) was consulted to supervise and verify the results. Only prostate cancer areas were segmented, since PCa was the only disease affecting the patients. All the segmented slices formed the volume of interest (VOI).

At this point, matRadiomics [28], which integrates the Pyradiomics [29] extractor, was used to extract 1781 features from PET images. Previously, PET images were converted into standardized uptake value (SUV) images in such a way that the radiomics features considered the normalization of the voxel activity. Radiomics features were then extracted both from non-pre-processed images (original images) and from pre-processed images after the application of wavelet decomposition and Laplacian of Gaussian (LoG) filtering. Overall, 107 features belonged to original images, 930 to LoG images and 744 to wavelet decomposed images (see Fig. 1). Features could be grouped in three categories: (i) shape features; (ii) first-order statistics features; (iii) texture features. Moreover, texture features could be grouped in the gray level co-occurrence matrix (GLCM), gray level run length matrix (GLRLM), gray level size zone matrix (GLSZM), neighboring gray tone difference matrix (NGTDM), and the gray level dependence matrix (GLDM) [30–34].

For reproducibility, the complete Pyradiomics configuration used in matRadiomics is reported in Table 1. All the other parameters were left to Pyradiomics default.

**Table 1:** Pyradiomics feature extractor configuration. The other parameters are left to Pyradiomics default

Bin width	0.25
Isotropic voxel	2 × 2 × 2
Interpolator	SitkBSpline
Wavelet method	Coif1
LoG sigma	[0.5, 1, 1.5, 2, 2.5, 3, 3.5, 4, 4.5, 5]
Normalization	True; scale = 1

### Preliminary Machine Learning Analysis

A preliminary analysis was conducted to identify subsets of selected features, establish a pool of model hyperparameters, and determine the most effective classifiers among discriminant analysis (DA), support vector machines (SVM), *k-*nearest neighbors (KNN), neural networks (NN), random forest (RF), and AdaBoost (Boost). The findings emerged from this step were subsequently utilized to construct the final machine learning (ML) model.

The preliminary analysis involved a pipeline of 30 iterations comprising (i) stratified training/testing splitting (80/20 ratio) to ensure that the distribution of classes in both datasets reflects the overall distribution in the original dataset, (ii) feature selection using the least absolute shrinkage and selection operator (LASSO), (iii) fivefold cross-validation for hyperparameter Bayesian optimization and model validation, and (iv) model testing. To ensure maximum robustness of the proposed pipeline, each model was trained and validated using the same folds during the fivefold cross-validation.

At the end of each repetition (30 in total), a subset of selected features and six optimized models (i.e., DA, SVM, KNN; NN, RF, and Boost) were obtained. In this way, a total of 30 subsets of selected features and 180 models were built at the end of the pipeline.

The frequency of each selected feature across pipeline repetitions was computed as follows:

$$featur{e}_{frequency}=\frac{n}{rep}\times 100,$$

where *n* represents the number of times a feature was selected (with a maximum equal to *rep*) and rep denotes the total number of pipeline repetitions (30 in this instance).

Finally, models’ performance of each classifier was averaged over the 30 repetitions to obtain the average accuracy, area under curve (AUC), sensitivity, specificity, precision, and *f-*score metrics. The models that obtained the highest average metrics were further considered for the construction of the final ensemble ML model.

**Fig. 2:**
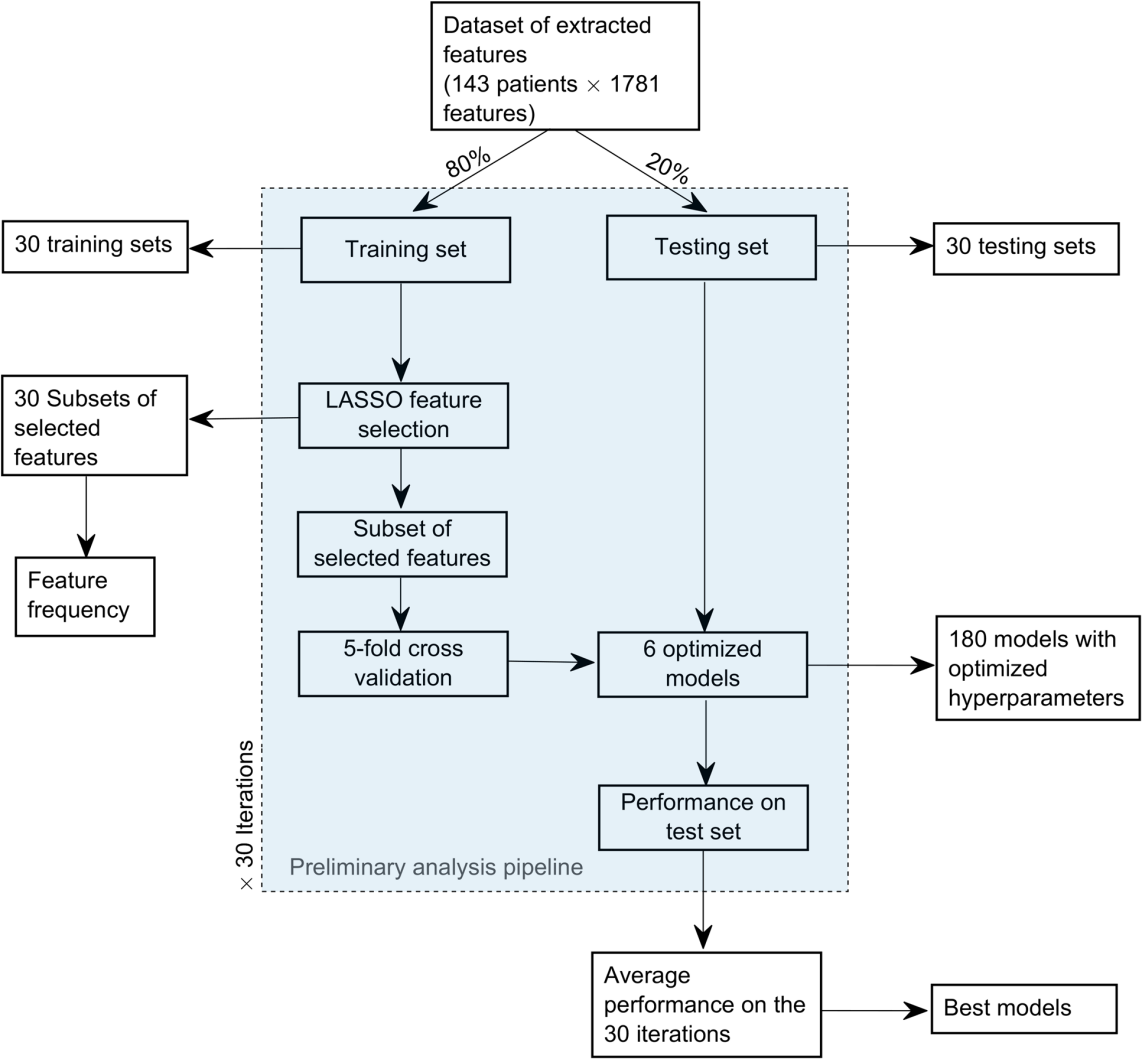
Preliminary analysis pipeline design. The pipeline is iterated 30 times to identify the best model candidates for the ensemble model, generate subsets of selected features, and optimize hyperparameters

The workflow of the preliminary analysis is illustrated in Fig. 2.

### Construction of the Ensemble Machine Learning Model

Starting from what emerged from the preliminary analysis, the best-performing models were identified as the candidates to build the ensemble ML model. Then, the hyperparameters of each candidate model were set, considering those obtained in the preliminary analysis. Since the individual classifiers were optimized across 30 repetitions, as outlined in the preliminary analysis pipeline, the set of hyperparameters could have varied across iterations. Therefore, a careful selection of the best model hyperparameters was carried out.

To identify the optimal subset of selected features, 11 subsets were initially generated based on their features frequency. These subsets were created as follows: the first subset consisted of features achieving a ${\text{feature}}_{\text{frequency}}\ge 90\%$ (namely, “*features90*” subset), the second subset included features with a ${\text{feature}}_{\text{frequency}}\ge 80\%$ (namely, “*features80*” subset), the third subset contained features with a ${\text{feature}}_{\text{frequency}}\ge 70\%$ (namely, “*features70*” subset). Following the construction of these initial three subsets, the remaining eight subsets were created by decreasing the threshold by ten percentage points (range 90–0%). It is worth noting that 0% indicates that all features were considered. Finally, an additional threshold of 5% was introduced, resulting in a total of 11 subsets.

Furthermore, two additional subsets of selected features were generated, namely “*finetuning*” and “*features7030r*” subsets. The "*finetuning*” subset is obtained by considering the features with a ${\text{feature}}_{\text{frequency}}$ between 30 (included) and 70 (not included) that were not correlated with the “*features70*” subset. Correlation was measured using the Pearson correlation coefficient (PCC) with a threshold of 0.3. The average PCC over the 30 iterations was considered as final value. The “*features7030r*” subset was the union between the “*finetuning*” and “*features70*″ subsets.

**Fig. 3:**
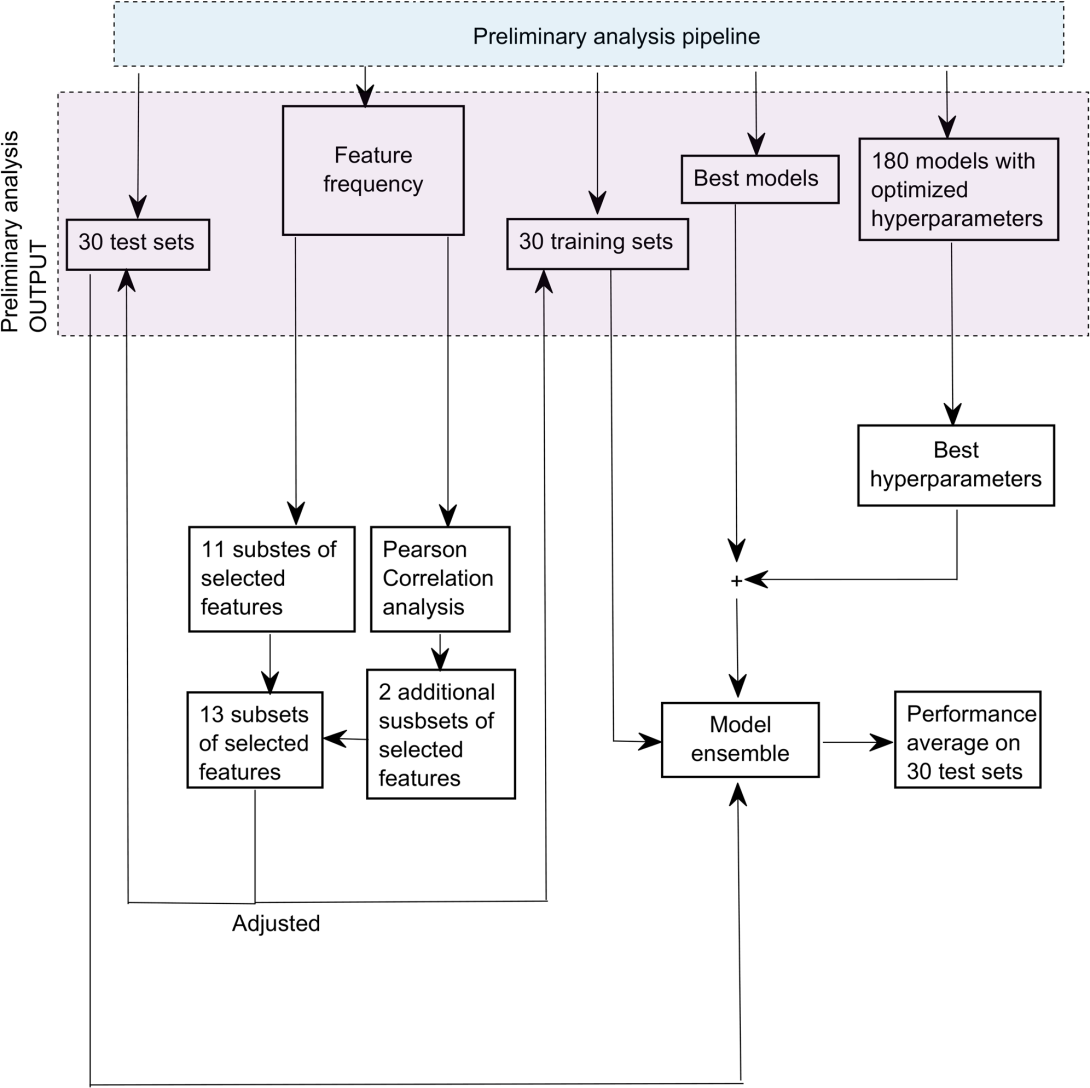
Flow chart from preliminary analysis to the ensemble model and the individuation of the two additional subsets

Overall, 13 subsets of selected features were generated. Figure 3 shows the workflow from preliminary analysis to the construction of the ensemble ML model.

Ensemble ML model predictions were obtained through majority voting, i.e., if an observation was labelled as “high–risk” by the majority of models constituting the ensemble, the observation was labelled as “high-risk.” The same consideration for the “low-risk” label. To obtain the AUC of the ensemble ML model, the weighted average posterior probability that one observation was “high-risk” or “low-risk” was calculated as follows:

$${w}_{\text{probability}}=\frac{\sum_{i=1}^{n}{w}_{i}\times {p}_{i}}{\sum_{i=1}^{n}{w}_{i}},$$

where $n$ is the number of models that constitute the ensemble ML model, ${w}_{i}$ is the AUC of the *i*th model, and ${p}_{i}$ is the posterior probability that the *i*th model assigned to that observation. Then, the receiver operating characteristic (ROC) curves was built using the label predictions and the weighted posterior probabilities.

Model performance was evaluated using the same test set subdivisions generated in the preliminary analysis. The performance was then averaged over 30 repetitions.

### Statistical Analysis

To verify if the different ensemble models, based on the 13 subsets of selected features, obtained statistically significant differences in performance metrics over the 30 iterations, we used the Friedman test, followed by post hoc tests corrected with Dunn-Sidak correction for multiple comparisons. All data related to ensemble models are presented as mean and 95% confidence interval (CI). MATLAB R2023b was used to perform the analysis [35]. A double-sided *p* value less than 0.05 was considered statistically significant.

## Results

### Characteristics of the Included Patients

Among 832 patients who underwent [^18^F]-PSMA-1007 PET/CT imaging, only 143 patients met the inclusion criteria, including 72 high-risk patients and 71 low-risk patients. The average age was 68.8 ± 8.6, 48 patients were characterized by a GS = 6, 56 by a GS = 7, 15 by a GS = 8, 23 by a GS = 9, and one patient by GS = 10. Overall, 39 high-risk and 36 low-risk patients were scanned using the Siemens equipment, while 33 high-risk and 35 low-risk patients with the GE equipment. The characteristics are reported in Table 2. Finally, patients with a Gleason score equal to 8, 9, and 10 was binned together to the patients who got a GS score equal to 4 + 3 (GS = 7), thus leading to two groups of patients: the low-grade (GS = 3 + 3, GS = 3 + 4) and high-grade (GS = 4 + 3, 4 + 4, 4 + 5 and 5 + 5) groups.

**Table 2:** Table with patients’ characteristics

Variable	Value
Patients’ age (mean ± std)	68.8 ± 8.6
High-risk patients	72
Low-risk patients	71
GS = 6	48
GS = 7	56 (3 + 4: 23, 4 + 3: 33)
GS = 8	15
GS = 9	23
GS = 10	1
Scanned by GE	33 high risk, 35 low risk
Scanned by Siemens	39 high risk, 36 low risk

### Results of the Preliminary Machine Learning Analysis

**Fig. 4:**
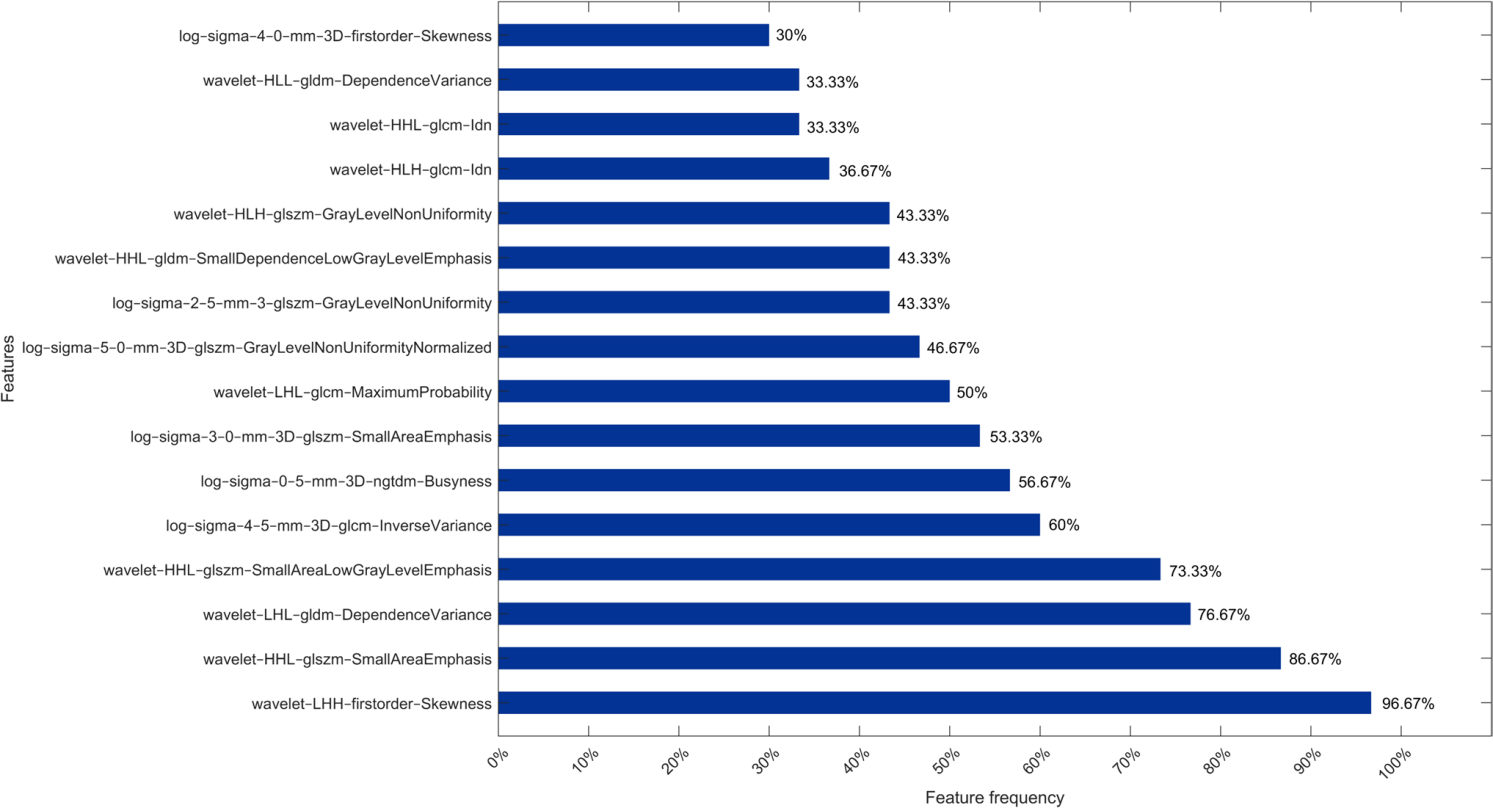
Selected features obtaining a frequency ranging from 30 to 100%

After the iteration of the preliminary analysis pipeline, 30 subsets of selected features were generated, which differed in size and type of features. Overall, subset sizes ranged from five to 25 features for a total of 79 features selected among the 1781 initial features. Among the 79 features, only one feature, namely “*wavelet*-*LHH*-*firstorder*-*Skewness*,” obtained a ${\text{feature}}_{\text{frequency}}$ greater than 90% (96.67%), while four features obtained a ${\text{feature}}_{\text{frequency}}$ greater than 70%, namely “*wavelet*-*LHH*-*firstorder*-*Skewness*” (96.67%), “*wavelet*-*HHL*-*glszm*-*SmallAreaEmphasis*” (86.67%), “*wavelet*-*LHL*-*gldm*-*DependenceVariance*” (76.67%), and “*wavelet*-*HHL*-*glszm*-*SmallAreaLowGrayLevelEmphasis*” (73.33%). The features that obtained a ${\text{feature}}_{\text{frequency}}$ greater or equal to 30% are reported in Fig. 4. In summary, 43 features belonged to the wavelet decomposed images, 33 to LoG filtered images, and three to original images. For what concerning feature classes, 18 features belonged to the GLCM class, two to the GLRLM class, 33 to the GLSZM class, nine to the GLDM class, three to the NGTDM class, and 14 to the first-order feature class, as reported in Table 3.

**Table 3:** Composition of the 79 selected features divided by image types and feature classes

Image type	Shape	First Ooder	GLCM	GLRLM	GLSZM	GLDM	NGTDM
Original	0	1	0	1	1	0	0
Wavelet	0	9	15	0	12	5	2
LoG	0	4	3	1	20	4	1

As result of this preliminary analysis on test sets, the NN classifier reached the highest average accuracy (71.67%) and specificity (74.05%), the DA classifier reached the highest average sensitivity (71.67%) and *f*-score (70.85%), while the SVM classifier reached the highest average AUC (76.26%) and precision (71.28%). Therefore, NN, DA, and SVM were individuated as the best candidates to build the ensemble ML model.

### Ensemble Machine Learning Model Performance

As reported in the previous analysis, DA, SVM, and NN were individuated to build the ensemble ML model.

For the first classifier, the most frequent kernels were “*linear*,” “*diaglinear*,” and “*pseudolinear*.” For each kernel, the amount of regularization (gamma hyperparameter) and linear threshold coefficient (delta hyperparameter) were set to (0.3354, 0.2081), (0.4298, 0.1525), and (0.3722, 0.2536), respectively.

For the SVM classifier, the most frequent kernel was the “*gaussian*” kernel, and the box constraint hyperparameter was set to 984.2237.

For the NN classifier, the size of the last fully connected layer was set to 1, the “sigmoid” was chosen as activation function, the weights of the fully connected layer were initialized through the He initializer [36], and the initial fully connected biases were set to zeros.

**Fig. 5:**
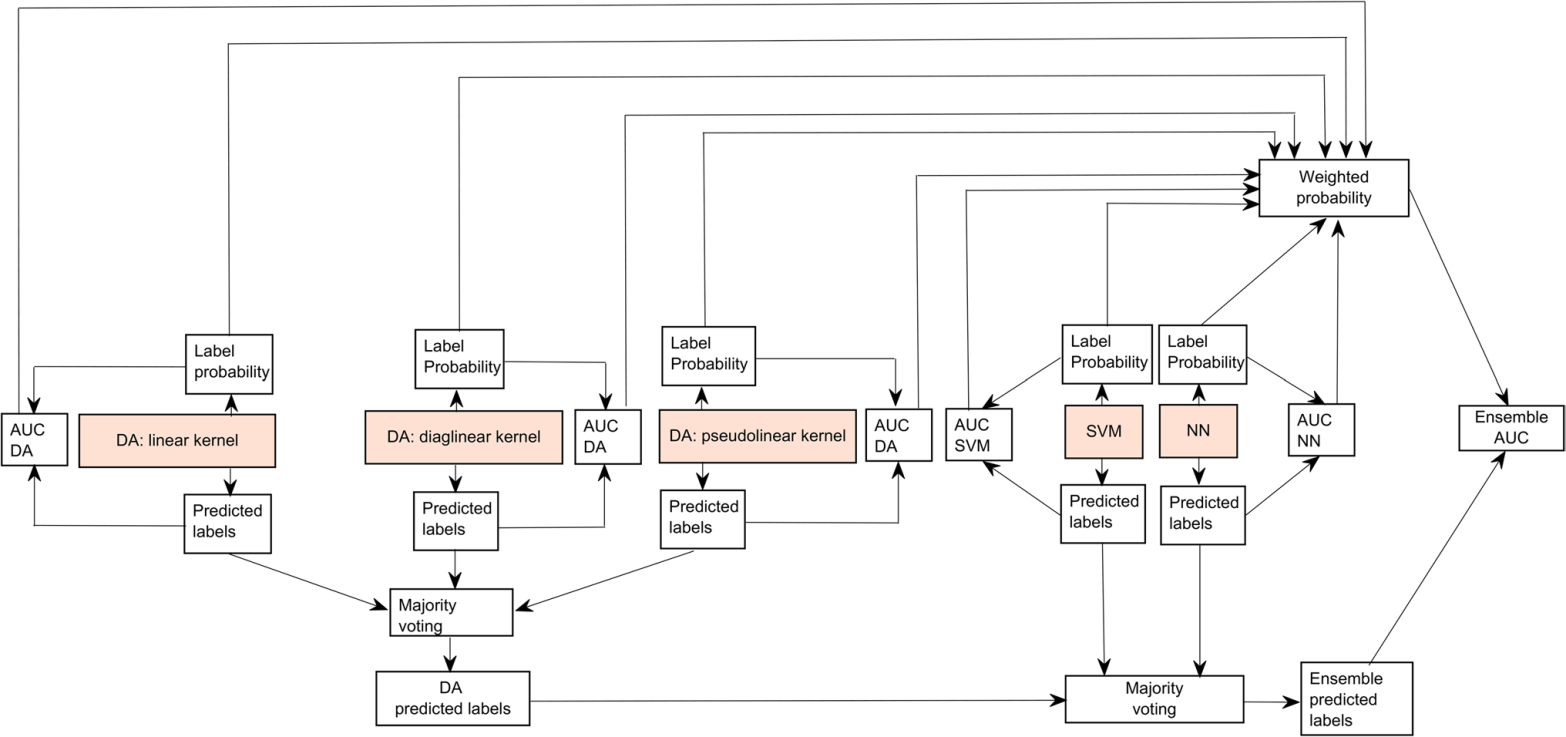
Ensemble ML model design. The classifiers constituting the model are highlighted in orange

The tables showing the full selected hyperparameters are reported in the Supplementary Material 1 file (Tables SM–1.1, SM–1.2, SM–1.3). Therefore, five models (three based on different DA kernels, one on SVM and one on NN) were used to build the ensemble ML model. The final model is shown in Fig. 5.

It was trained using the 13 feature subsets described in “Construction of the ensemble machine learning model” section and reported in Table 4 with their characteristics. Regarding the “*finetuning*” dataset, only seven features, among the 12 features that had a frequency in a range between 70% (not included) and 30% (included), were retained after the correlation analysis. Therefore, the “*finetuning*” dataset comprised seven features: (i) “*log****-****sigma****-****0****–****5****-****mm****-****3D****-****ngtdm****-****Busyness*,” (ii) “*log****-****sigma****-****2****–****5****-****mm****-****3D****-****glszm****–****GrayLevelNonUniformity*,” (iii) “*wavelet****-****HHL****-****gldm****-****SmallDependenceLowGrayLevelEmphasis*,” (iv) “*wavelet****-****HLH****-****glszm****-****GrayLevelNonUniformity*,” (v) “*wavelet****-****HLH****-****glcm****-****Idn*,” (vi) “*wavelet****-****HHL****-****glcm****-****Idn*,” (vii) “*log****-****sigma****-****4****–****0****-****mm****-****3D****-****firstorder****-****Skewness*.” In Table 5, we reported the average PCC values that each of the 12 features obtained and highlighted the seven features that obtained a PCC < 0.3. The feature that obtained the lowest PCC (0.0948) was the “*log-sigma-2–5-mm-3D-glszm-GrayLevelNonUniformity*.”

**Table 4:** Datasets of selected features used to train the ensemble ML model, with their specifications and their sizes

Dataset name	Feature frequency	Subset size
features90	≥ 90%	1
features80	≥ 80%	2
features70	≥ 70%	4
features60	≥ 60%	5
features50	≥ 50%	8
features40	≥ 40%	12
features30	≥ 30%	16
features20	≥ 20%	23
features10	≥ 10%	34
features5	≥ 5%	52
features0	All 79 features	79
features7030r	Union between finetuning and features70 subsets	11
finetuning	fine**-**tuning subset	7

**Table 5:** Features with a frequency in a range from 70 to 30%, together with their average PCC value and feature frequency. Bold is used to highlight features that got a PCC < 0.3 and were included in the fine**-**tuning subset

Feature	Average PCC	Feature frequency
log**-**sigma**-**4**–**5**-**mm**-**3D**-**glcm**-**InverseVariance	0.3369	60%
**log-sigma-0–5-mm-3D-ngtdm–Busyness**	**0.2735**	**56.67%**
log**-**sigma**-**3**–**0**-**mm**-**3D**–**glszm**–**SmallAreaEmphasis	0.3756	53.33%
wavelet**-**LHL**-**glcm**-**MaximumProbability	0.3758	50%
log**-**sigma**-**5**–**0**-**mm**-**3D**-**glszm**–**GrayLevelNonUniformityNormalized	0.3322	46.67%
**log-sigma-2–5-mm-3D-glszm-GrayLevelNonUniformity**	**0.0948**	**43.33%**
**wavelet-HHL-gldm-SmallDependenceLowGrayLevelEmphasis**	**0.1595**	**43.33%**
**wavelet-HLH-glszm–GrayLevelNonUniformity**	**0.1341**	**43.33%**
**wavelet-HLH-glcm–Idn**	**0.1833**	**36.67%**
**wavelet-HHL-glcm–Idn**	**0.2470**	**33.33%**
wavelet**-**HLL**-**gldm**-**DependenceVariance	0.3301	33.33%
**log-sigma-4–0-mm-3D-first-order-Skewness**	**0.1659**	**30%**

**Fig. 6:**
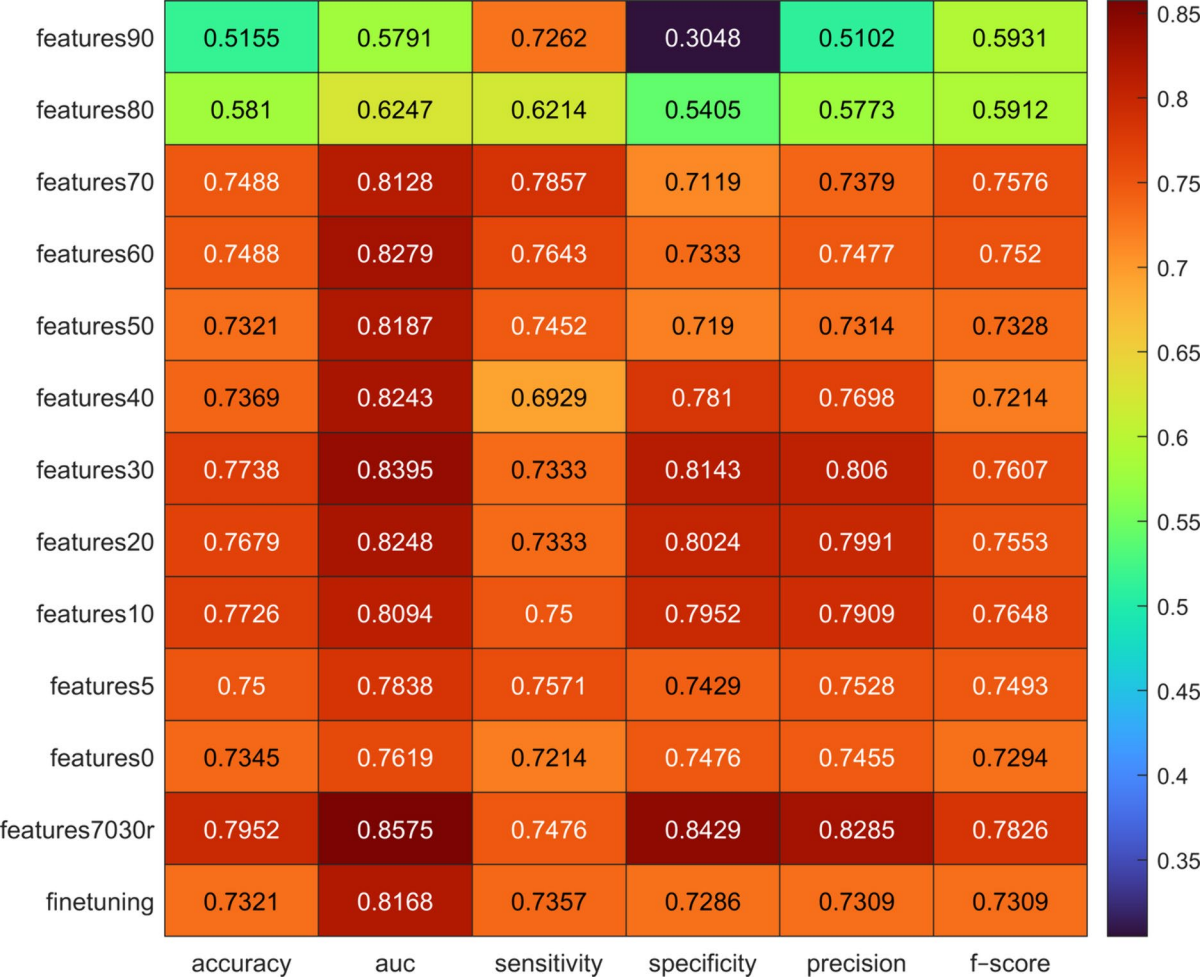
Performance of the ensemble ML model at variations of feature datasets. Intense red represents the best performance, while intense blue the lowest performance. In this heatmap, only the average value is represented

**Fig. 7:**
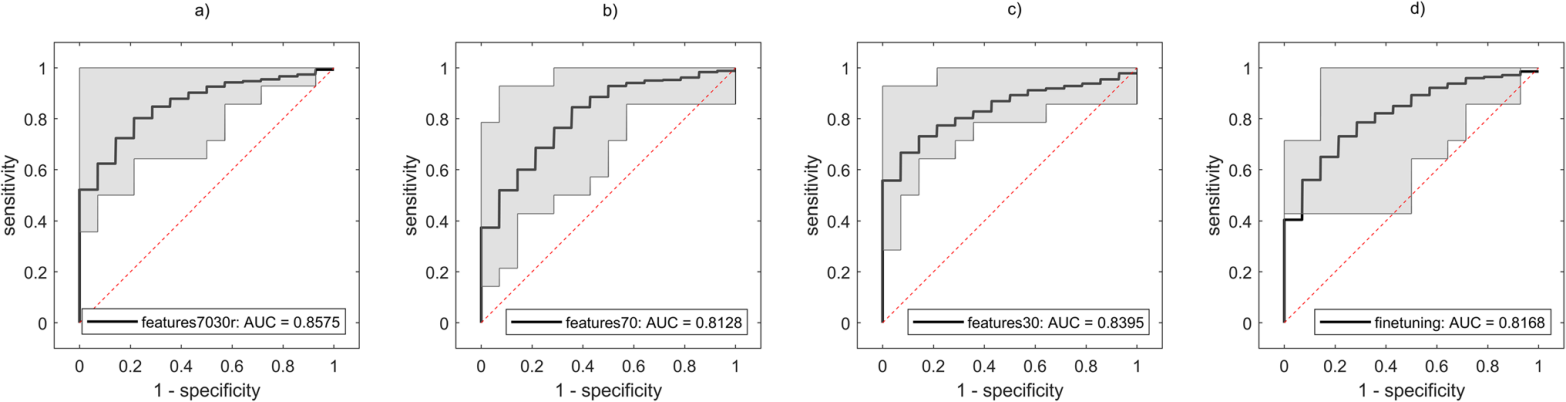
ROC comparison between: **a** “*features7030r*,” **b** “*features70*,” **c** “*features30*,” **d** “*finetuning*” datasets. The average ROC curves are represented in black, while the gray-shaded areas represent the areas between the ROCs with the minimum AUC and the ROCs with the maximum AUC

Finally, the performance of the ensemble ML model was computed at variations of feature datasets as shown in Fig. 6. The model trained with the “*features7030r*” dataset obtained the highest average accuracy (79.52%, 95%CI 77.38–81.79%), AUC (85.75%, 95%CI 83.36–87.79%), specificity (84.29%, 95%CI 81.67–84.43%), precision (82.85%, 95%CI 80.53–85.17%), and *f*-score (78.26%, 95%CI 75.74–80.87%) on test sets, while the highest average sensitivity (78.57%, 95%CI 74.76–81.90%) was reached by the model ensemble trained with the “*features70*” dataset, as shown in Table 6. Moreover, a comparison between ROC curves is shown in Fig. 7.

**Table 6:** Performance of the model ensemble at variations of feature datasets expressed as mean and 95%CI (between brackets). Best performance is highlighted in bold

Dataset	Accuracy	AUC	Sensitivity	Specificity	Precision	*F*-score
features90	51.55% (48.79–53.57%)	57.91% (54.02–61.27%)	72.62% (66.67–79.29%)	30.48% (25.48–35.95%)	51.02% (49.04–52.67%)	59.31% (56.34–61.92%)
features80	58.10% (55.36–60.95%)	62.47% (59.62–65.28%)	62.14% (57.14–67.86%)	54.05% (48.57–58.32%)	57.73% (55.16–60.64%)	59.12% (55.67–62.23%)
features70	74.88% (72.02–77.86%)	81.28% (78.43–84.10%)	**78.57%** (74.76–81.90%)	71.19% (66.67–75.71%)	73.79% (70.81–77.50%)	75.76% (73.00–78.46%)
features60	74.88% (71.50–77.31%)	82.79% (80.12–85.27%)	76.43% (72.38–80.00%)	73.33% (68.57–76.90%)	74.77% (71.27–77.64%)	75.20% (71.88–77.61%)
features50	73.21% (70.71–75.71%)	81.87% (79.37–84.76%)	74.52% (70.24–78.44%)	71.90% (68.33–75.24%)	73.14% (70.48–75.96%)	73.28% (70.42–76.03%)
features40	73.69% (70.83–76.28%)	82.43% (79.95–84.78%)	69.29% (64.97–74.05%)	78.10% (73.33–81.90%)	76.98% (73.33–80.27%)	72.14% (69.10–75.13%)
features30	77.38% (75.36–79.67%)	83.95% (81.79–86.38%)	73.33% (69.29–77.51%)	81.43% (78.43–84.52%)	80.60% (78.01–83.64%)	76.07% (73.43–78.68%)
features20	76.79% (73.69–79.64%)	82.48% (79.98—85.06%)	73.33% (68.04–77.62%)	80.24% (75.76–83.81%)	79.91% (76.06–83.28%)	75.53% (71.71–78.57%)
features10	77.26% (74.52–79.76%)	80.94% (78.23–83.44%)	75.00% (70.71–78.81%)	79.52% (75.71–82.62%)	79.09% (75.73–81.71%)	76.48% (73.64–79.14%)
features5	75.00% (72.74–77.02%)	78.38% (75.66–81.07%)	75.71% (71.90–79.29%)	74.29% (70.24–77.38%)	75.28% (72.68–77.56%)	74.93% (72.57–77.34%)
features0	73.45% (71.19–76.07%)	76.19% (73.27–79.42%)	72.14% (68.81–75.95%)	74.76% (71.67–78.33%)	74.55% (71.85–77.36%)	72.91% (70.48–75.53%)
features7030r	**79.52%** (77.38–81.79%)	**85.75%** (83.36–87.79%)	74.76% (71.19–78.57%)	**84.29%** (81.67–86.43%)	**82.85%** (80.53–85.17%)	**78.26%** (75.74–80.87%)
finetuning	73.21% (69.90–76.45%)	81.68% (78.21–84.46%)	73.57% (69.52–78.10%)	72.86% (69.36–76.43%)	73.09% (69.81–76.11%)	73.09% (69.64–76.64%)

**Fig. 8:**
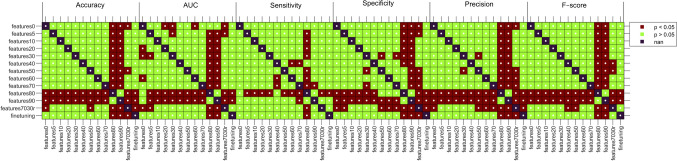
Statistical difference in performance between models expressed through *p-*value results. Intense red is used to highlight cases where the *p*-value is less than 0.05, indicating statistical significance, while light green is employed for cases where the *p*-value is greater than 0.05, suggesting no significant difference

Statistical differences in accuracy, AUC, specificity, precision, and *f*-score values between models were calculated. Figure 8 illustrates the results based on p-values.

A further interesting result is that each feature of the “*features7030r*” subset, if taken alone, showed an average accuracy always below 70%, an average AUC always below 70%, except for the “*wavelet-LHL-gldm-DependenceVariance*” feature (74.71%), an average precision always below 70%, and an average *f*-score always below 70% apart from the “*wavelet-LHL-gldm-DependenceVariance*” (71.67%).

## Discussion

The aim of this study was to develop a robust ensemble machine learning model capable of differentiating “high-risk” and “low-risk” prostate cancer, and to investigate the impact of radiomics features on the final trained model.

The [^18^F]-PSMA tracer PET/CT plays an important role in pretreatment setting and high-risk PCa monitoring. However, the visual method is subjective, and there may be abnormal uptake of PSMA both in PCa lesions and benign lesions, and this can lead to difficulties in reporting. Radiomics analyses promise the benefit of automation, which can reduce human error and prevent unnecessary biopsies associated with misdiagnosis. The role of AI decision systems will evolve particularly as the AI systems become more accurate.

Starting from the hypothesis that a robust model requires robust features capable of maintaining stability across diverse training datasets, a preliminary analysis pipeline was developed to identify the most frequent radiomics features and the best candidate models for the construction of the ensemble model. Among the 79 most frequent features, no shape features were selected. They belonged to the wavelet decomposed images (43 feature in total), LoG filtered images (33 features in total), and only three to the original images. Successively, we grouped the 79 features in 11 subsets based on their ${\text{feature}}_{\text{frequency}}$.

Regarding the best candidate models, DA (constituted by three sub-models), SVM and NN models were identified to build the model ensemble. To maintain the consistency of the proposed model, the ensemble model was trained and tested using the same training and testing sets generated in the preliminary analysis. As result, the “*features70*” subset obtained the highest sensitivity, while the “*features30*” subset obtained the highest accuracy, AUC, specificity, and precision. To leverage the obtained results without sacrificing feature stability, we added two additional feature subsets (namely, “*features7030r*” and “*finetuning*”), for a total of 13 subsets. In other words, to reach a good compromise between the results obtained with the “*features70*” and “*features30*” subsets, the “*features70*” subset were combined with the features with a $70\%< {\text{feature}}_{\text{frequency}}\le 30\%$, and the Pearson correlation analysis was used to reduce the presence of less stable features, identifying the “*finetuning*” dataset. The combination of the “*features70*” and “*finetuning*” datasets led to the creation of the “*features7030r*” dataset. We analyzed the performance of the model ensemble with these two new datasets and observed that the “*features7030r*” subset outperformed all feature subsets, except for the sensitivity value (78.57% for *features70* and 74.76% for *features7030r)*. Moreover, upon examining the individual performance of the 79 features, along with that of the “*features90*” (displaying the lowest performance) and “features80” (exhibiting the second lowest performance) datasets, we deduce that the synergy between robust features and fine-tuning features significantly enhanced the performance of the model ensemble. Notably, this ensemble also surpassed the average performance achieved by the classifiers in the preliminary analysis. Moreover, no original features appeared in the “*features7030r*” dataset, showing how image pre–processing could enhance relevant high-frequency details in the image, and reduce image noise, leading to the most predictive radiomics features [25, 37, 38].

In literature, two studies [15, 16] showed the potential of radiomics in differentiating between high-risk and low-risk prostate cancer patients undergoing [^18^F]-PSMA-1007 PET/CT imaging. In the study [15], a combined radiomics and clinical nomogram was proposed with a maximum AUC, sensitivity, specificity, and precision of 71.90%, 47.1%, 81.3% and 74.30% respectively, significantly lower than those obtained by us (85.75%, 74.76%, 84.29%, and 82.85%, respectively). In [16], the authors investigated the performance of radiomics at variations of volume segmentation thresholds, finding that one of the most robust features to threshold variations was the “*firstorder*-*skewness*” feature. Also in our study, the “*skewness*” feature was included in the best performing feature subset, even though related to wavelet and LoG filtered images. The authors in [16] used the SVM classifier reaching a maximum accuracy, AUC, sensitivity, specificity, and *f*-score of 73%, 81%, 91%, 35% and 82%. In comparison, our model showed greater accuracy (79.52%), AUC (85.75%), precision (82.85%), and specificity (84.29%), while lower *f*-score (78.26%) and sensitivity (74.76%) [14].

In addition, our findings underscore the importance of utilizing a model ensemble [39] to ensure more robust performance when compared to employing single classifiers. Furthermore, the fact that our model achieved a more balanced performance between sensitivity and specificity suggests its potential applicability across a wide range of scenarios. Finally, unlike previous studies, thanks to matRadiomics, the PET images were converted into SUV images, preserving crucial information that would otherwise be lost when Pyradiomics is used as a standalone feature extraction tool. However, none of the SUV-based values used in clinical settings (such as SUV_max_ or SUV_mean_) were selected among the 79 features. The only SUV based features that were selected, were those related to the intensity distribution of gray levels, namely “*wavelet-LHH-firstorder-Skewness*” and “*log-sigma-4–0-mm-3D-firstorder–Skewness*,” However, they are not commonly used in clinical settings, since based on skewness and pre-processed images. Moreover, the *wavelet-LHH-firstorder-Skewness* feature is part of the “*features 90*” dataset as the only feature and showed poor performance in risk stratification, as shown in Fig. 6. This aspect, together with the exclusion of SUV_max_ and SUV_mean_ from selected features, may indicate that SUV alone cannot be used to differentiate patients in low-risk and high-risk groups with good performance.

The proposed pipeline offers valuable insights into feature stability, an often-overlooked aspect in radiomics studies that is essential for small datasets. This focus on stability enhances model explainability and interpretation. Relying on a single train/test split can lead to biased outcomes; fortunate splits may produce overly optimistic results, while unfortunate ones could yield pessimistic results. The performance of models trained on small datasets is highly sensitive to these splits. In contrast, the combination of train/test splitting and cross-validation for model optimization, as implemented in our preliminary analysis, strengthens the study’s robustness. This approach not only facilitates the identification of the most promising candidate models through an objective criterion but also helps streamline the final ensemble model by reducing complexity. Additionally, we ensured that the same training and testing sets were utilized for the ensemble model, allowing for a fair comparison with the base models. Notably, our analysis indicates that the interplay between robust features and those with moderate stability enhances overall model performance. Starting from a comprehensive pool of selected features, we employed a non-subjective method to pinpoint the optimal features, refining the selection through Pearson correlation analysis to achieve an ideal subset.

The proposed study has several limitations, including its single-center design, the use of manual segmentation, the small cohort size, and the absence of an external test set. Multicenter studies should be preferred to enhance model generalization capabilities, and, as it was shown in previous studies [40, 41] not only related to prostate, manual segmentation is operator-dependent, time-consuming [42], and could have a negative impact on features reproducibility, even if it remains the gold-standard segmentation method in PET imaging. Moreover, gold standard was based on biopsy and not on postoperative pathology results, and lesions were considered positive based on the nuclear medicine specialist experience, which could lead to inaccuracies of results. Finally, future studies will aim to incorporate clinical variables alongside features extracted from other imaging modalities into the radiomics workflow.

## Conclusion

This study provided a meticulous analysis on the development of a robust radiomics ensemble model capable of differentiating between “high-risk” and “low-risk” prostate cancer and showed that the interaction between robust features and fine-tuning features led to the highest average accuracy (79.52%), AUC (85.75%), specificity (84.29%), precision (82.85%), and *f*-score (78.26%). As result, [^18^F]-PSMA-1007 PET radiomics could potentially enhance PCa risk stratification without having to rely exclusively on biopsies. Furthermore, by providing the trained ensemble model, we guaranteed its immediate re-use for future predictions and further external validation.

## Supplementary Information

Below is the link to the electronic supplementary material.

Supplementary file1 (DOCX 14 KB)

## Data Availability

The repository which contains the code is available at the following URL: 10.5281/zenodo.11125273.
