# Supplementary material for: A Robust [18F]-PSMA-1007 Radiomics Ensemble Model for Prostate Cancer Risk Stratification
Source: J Imaging Inform Med. 2024 Sep 30;38(3):1388–402. doi: 10.1007/s10278-024-01281-w (PMC12092872; doi:10.1007/s10278-024-01281-w)
Supplement: Supplementary file 1 — Supplementary file1 (DOCX 14 KB) [file 10278_2024_1281_MOESM1_ESM.docx]

**Supplementary Material 1**

This supplementary material file is related to the chosen hyperparameters for the different models used in the final ensemble, as described in the manuscript.

Table SM–1.1: The chosen hyperparameters for the DA classifier

| Kernel function | gamma | delta |
| --- | --- | --- |
| linear | 0.3354 | 0.2081 |
| diaglinear | 0.4298 | 0.1525 |
| pseudolinear | 0.3722 | 0.2536 |

Table SM–1.2: The chosen hyperparameters for the SVM classifier

| Kernel function | Box constraint | Kernel scale |
| --- | --- | --- |
| gaussian | 984.2237 | 92.50 |

Table SM–1.3: The chosen hyperparameters for the NN classifier

| Fully connected layer size | Activation | Weight initializer | Bias Initializer |
| --- | --- | --- | --- |
| 1 | sigmoid | he | zeros |
